# Simultaneous unilateral central retinal vein occlusion and branch retinal artery occlusion after Coronavirus Disease 2019 (COVID-19) mRNA vaccine

**DOI:** 10.5935/0004-2749.2022-0010

**Published:** 2023-03-08

**Authors:** Oscar Andree García Ruiz, Julio José González-López

**Affiliations:** 1 Hospital Universitario Ramón y Cajal, IRYCIS, Madrid, Spain

**Keywords:** COVID-19 vaccines/adverse effects, RNA, messenger, Retinal vein occlusion/diagnosis, Retinal artery occlusion, Hu-mans, Case report, Vacina contra COVID-19/efeitos adversos, RNA mensageiro, Oclusão da veia retiniana/diagnóstico, Oclusão da artéria retiniana, Humanos, Relato de casos

## Abstract

A 51-year-old non-obese woman presented with a one-week history of progressive blurry vision within the inferior visual field of her left eye. Her only relevant past medical history was long-standing hypothyroidism and recent vaccination against Coronavirus Disease 2019 (COVID-19) with an mRNA vaccine 12 days before the onset of symptoms. At examination, the anterior segment was unremarkable, but the retinal fundus revealed a central retinal vein occlusion associated with a branch retinal artery occlusion of the superior temporal branch in her left eye. Ancillary tests to rule out thrombophilia, hyperviscosity, hypercoagulability, or inflammation were negative. Ultrasound tests were also negative for a cardiac or carotid origin of the branch retinal artery occlusion. At two-month follow-up, no new retinal vascular occlusive events were observed. Although the best-corrected visual acuity at presentation was 8/10 in the left eye, the final best-corrected visual acuity remained 3/10.

## Abstract (pt)

Uma mulher de 51 anos, não obesa, apresentou história de uma semana de visão embaçada progressiva no campo visual inferior do olho esquerdo. Seu único histórico médico anterior relevante era hipotireoidismo de longa data e uma recente vacinação contra a Doença de Coronavírus 2019 (COVID-19), com vacina de mRNA, 12 dias antes do início dos sintomas. O exame mostrou segmento anterior normal, mas o fundo da retina revelou uma oclusão da veia central da retina associada a uma oclusão de ramo arterial da retina do ramo temporal superior no olho esquerdo. Testes auxiliares para descartar trombofilia, hiperviscosidade, hipercoagulabilidade ou inflamação apresentaram resultados negativos. Testes de ultrassom também foram negativos quanto a uma origem cardíaca ou da carótida da oclusão do ramo da artéria da retina. Após dois meses de acompanhamento, nenhum novo evento vascular oclusivo retiniano foi observado. Embora, a acuidade visual melhor corrigida na apresentação tenha sido de 8/10 no olho esquerdo, a acuidade visual final melhor corrigida permaneceu em 3/10.

## INTRODUCTION

We read with great interest the article recently published by da Silva et al. on retinal vascular findings after Coronavirus Disease 2019 (COVID-19) vaccination^(1)^. Similar to the cases described, we recently observed a woman with a simultaneous unilateral retinal artery and vein occlusion after a second dose of mRNA COVID-19 vaccine (Moderna-Lonza).

Simultaneous combined retinal artery and vein occlusions are uncommon and have been reported in patients with systemic lupus erythematosus, Behçet’s disease, ocular trauma, hyperhomocysteinemia, and leukemia^(2)^.

Systemic vascular thrombotic events are a well-des-cribed complication of COVID-19. Such events have been attributed to the hypercoagulable state induced by coronavirus infection^(2)^. Retinal vascular occlusions have also been observed during COVID-19 infection^(3-5)^. However, retinal vascular occlusive events have also been reported after administration of COVID-19 vacci-nes, both those based on adenoviral vectors and the ones based on mRNA^(6,7)^.

Therefore, we report a patient who had a retinal vascular occlusive event 12 days after receiving the second dose of the Moderna-Lonza vaccine, an mRNA COVID-19 vaccine.

## CASE REPORT

A 51-year-old non obese woman of Spanish descent presented at our university hospital casualty with chief complaint of blurry vision in her left eye for the last 1 week. She described the appearance of “grey patches” in her left inferior visual field which gradually increased in size after the onset.

Her past medical history included a recent SARS-CoV-2 vaccination with a mRNA vaccine (Moderna-Lonza^®^). The second dose was administered 12 days prior to the onset of symptoms. Besides, she had a history of long-standing hypothyroidism treated with levothyroxine. She denied any other concomitant diseases or consumption of any other drug.

**Figure 1:**
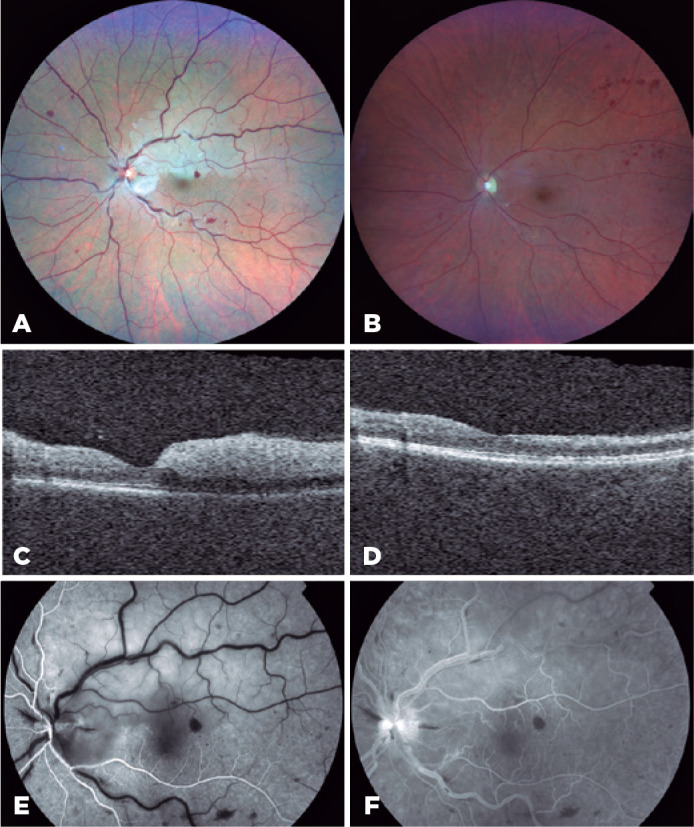
Multimodal imaging of retinal changes in a patient with simultaneous left eye central retinal vein occlusion and branch retinal artery occlusion following the second dose of the Moderna-Lonza Coronavirus Disease 2019 (COVID-19) mRNA vaccine. (A) Colour fundus photograph of the left eye at presentation showing disc swelling, diffuse venous tortuosity, blot and dot retina hemorrhages, and retinal whitening along the superotemporal artery tract. (B) Colour fundus photograph of the left eye two months after the onset of symptoms showing resolution of the disc swelling and retinal whitening, and superotemporal blot and dot hemorrhages. (C) Vertical optical coherence tomography scan of the left fovea at presentation, showing retinal edema with disorganization of retinal inner layers in the superior hemimacula. (D) Vertical optical coherence tomography scan of the left fovea at 2 months showing inner retinal atrophy in the superior hemimacula. (E) Arterial time fundus fluorescein angiography of the left eye at presentation showing delayed perfusion of the superotemporal retinal artery. (F) Late venous time fundus fluorescein angiography of the left eye at presentation showing delayed perfusion of the superotemporal retinal artery, a hot disc, venous tortuosity, and retinal hemorrhages.

At presentation, her best-corrected visual acuity (BCVA) was 10/10 Snellen in the right eye and 8/10 in the left eye. Anterior segment examination was unremarkable in both eyes. The intraocular pressure was 15 mmHg in the right eye and 16 mmHg in the left eye. Fundus examination was normal in her right eye, but the left eye showed optic disc swelling associated with peripapillary flame-shaped hemorrhages and widespread dot and blot retinal hemorrhages, together with retinal whitening along the course of the superior temporal arterial branch (Figure 1A). Optical coherence tomography (OCT) confirmed retinal edema with disorganization of retinal inner layers (DRIL) within the macula (Figure 1C). Fluorescence angiography confirmed an occlusion in the superior temporal branch of the retinal artery (Figure 1E) and a central retinal vein occlusion without cystoid macular edema (Figure 1F).

Blood pressure, echocardiography, and ultrasound examination of carotid arteries were normal. Hypercoagulability (lupus antiocoagulant, proteins C and S levels, factor V Leiden, prothrombin 20210A mutation, anticardiolipin antibodies), hyperviscosity workups (serum protein electrophoresis), and inflammation screening (erythrocyte sedimentation rate, C reactive protein) were within normal limits.

At one-month follow-up, no new episodes of vascular occlusion were reported. BCVA in the right eye was 10/10 and the BCVA in the left eye had declined to 3/10.

At two-month follow-up, no further vascular occlusive episodes were reported and BCVA was found to have remained stable. On fundus examination, there were still some widespread intraretinal hemorrhages in the left eye along with signs of retinal atrophy alongside the superior temporal branch of retinal artery (Figures 1B and D).

Different thromboembolic events have been reported after COVID-19 adeno-associated vaccine, which have been linked to antibodies against platelet factor 4^(8)^. In addition to retinal vascular events, ocular inflammation has also been recently described after COVID-19 vaccination^(9)^. To the best of our knowledge, this is the first case presenting with both central retinal vein occlusion (CRVO) and a branch retinal artery occlusion (BRAO) in the same eye after a second dose of an mRNA COVID-19 vaccine. Other potential causes were ruled out. Therefore, it would be interesting to know whether there could be a potential connection between the vaccine and the retinal vascular event. We agree with da Silva et al.^(1)^, that there is yet insufficient evidence to claim a causality link, as these cases may be explained by serendipity alone, given the fact that most of the population in Spain and Brazil has been exposed to COVID-19 vaccines during 2021. However, this potential adverse event should continue to be observed and reported.
